# Postnatal care services use by mothers: A comparative study of defaulters versus attendees of postnatal clinics in Enugu

**DOI:** 10.1371/journal.pone.0280315

**Published:** 2023-03-30

**Authors:** Chidinma Ifechi Onwuka, Euzebus Chinonye Ezugwu, Samuel Nnamdi Obi, Chidozie Onwuka, Cyril Chukwudi Dim, Chibuike Chigbu, Eric Asimadu, Ijeoma Victoria Ezeome, Tochukwu Christopher Okeke, Chukwuemeka Anthony Iyoke

**Affiliations:** 1 Department of Obstetrics and Gynaecology, Faculty of Medical Sciences, College of Medicine, University of Nigeria, Ituku-Ozalla, Enugu, Nigeria; 2 Oral and Maxillo-facial Surgery Department, King Khalid University, Abha, Saudi Arabia; Rivers State University Teaching Hospital, NIGERIA

## Abstract

**Introduction:**

Despite much emphasis on the reproductive health of women, maternal mortality is still high, especially in postnatal period.

**Objective:**

To assess the prevalence of postnatal care use and reasons for defaults among mothers attending the child immunization clinics in Enugu, Nigeria.

**Methods:**

This was a cross-sectional comparative study of 400 consecutive nursing mothers who presented at the Institute of Child Health of UNTH and ESUTH, Enugu for Second dose of the Oral Polio Vaccine (OPV2) for their babies at 10 weeks postpartum. Data was collected using Interviewer-administered questionnaire and subsequently analyzed with version 22.0 IBM SPSS software, Chicago, Illinois. A p-value of less than 0.05 was considered as statistically significant.

**Result:**

The prevalence of the 6th week postnatal clinic attendance among the mothers was 59%. The majority of the women (60.6%) who had antenatal care by skilled birth attendants attended postnatal clinic. Unawareness and being healthy were the main reasons for not attending postnatal clinic. Following multivariate analysis, place of antenatal (OR = 2.870, 95% C.I = 1.590–5.180, p < 0.001) and mode of delivery (OR = 0.452, 95% C.I = 0.280–0.728, p = 0.001) were the only significant predictors of postnatal clinic attendance (p < 0.05).

**Conclusion:**

Postnatal clinic attendance by women in Enugu is still suboptimal. The main reason for non-attendance of the 6th week postnatal clinic was lack of awareness. There is need for healthcare professionals to create awareness about the importance of postnatal care and encourage mothers to attend.

## Background

Postnatal care (PNC) is an important aspect of maternal health-care services not just for the mother but also the newborn [1]. It ensures the return to normal of some changes in pregnancy and delivery, as well as detection of other abnormalities [2]. A proper postnatal care service is a key strategy to reduce maternal and neonatal mortality [3]. Problems such as maternal infection, severe infection of newborn as well as inappropriate feeding practices could be reduced if women receive appropriate postnatal care [4]. Postnatal care will not only help in maintaining and promoting the health of the woman and the newborn baby; it also provides an opportunity for health professionals to identify, monitor and manage health conditions that may occur in the mother and newborn during the postnatal period [4]. Moreover, it also provides health care professionals with the opportunity to promote personal hygiene, appropriate feeding practices, exclusive breastfeeding, and family planning counseling and services as well as immunization of newborns [4]. Therefore, maternal and newborn health can be improved through proper postnatal care services under the care of skilled healthcare personnel [3].

Maternal mortality remains high in the developing world with over 60% of maternal death occurring during the postpartum period [5, 6]. Just like in other developing countries, high mortality rate in Nigeria has been linked to low use of maternal health services and postnatal care [7]. Postnatal care is one the interventions for reducing maternal and neonatal deaths [5]. Postnatal care also provides opportunity for mothers to reinforce their knowledge on best practices (such as support and information on exclusive breastfeeding, breast care, adequate nutrition, etc.) in motherhood initiated during the antenatal period [8]. Low coverage of care in the postnatal period affects other maternal, newborn and child health programmes negatively [5]. The long-term maternal complications due to lack of care in the postnatal period include chronic pelvic pain, damage to the reproductive system, impaired mobility and infertility [5].

Postnatal care services utilization can be affected by several factors which include maternal age, educational level of the women, occupational status of women and husbands, place of delivery, mode of delivery, number of pregnancies, attitude of the healthcare personnel, cost, awareness about obstetric related danger sign, and awareness about postnatal care services [1, 3, 5, 7, 9, 10].

Postnatal care (PNC) for the mother and infant has been a neglected area, even for women who give birth in a health facility [11]. It receives relatively less attention than pregnancy, labour and delivery and this has led to increased maternal morbidities and mortalities with resultant readmission of the women because of complications such as perineal wound infections, accidents, puerperal sepsis, thromboembolism, mental disorder, postpartum eclampsia and secondary postpartum haemorrhage [12].

Women in sub-Sahara Africa receive little or no postnatal care [6, 13]. These women, including the educated ones, default from the use of postnatal care services probably as a result of ignorance or feeling of being healthy [13, 14].

Although WHO recommends 4 PNC visits; the practice in Nigeria especially Enugu is skewed in favour of 6 weeks only [15]. The PNC service at 6 weeks is however still being underutilized thereby making postnatal care programs among the weakest of all reproductive and child health programs. Reported PNC services utilization in Nigeria range from 16.9% to 41.2% [7, 13, 16].

Though, there have been some studies on postpartum and postnatal care services utilization in Enugu; to the best of our knowledge there is paucity of data on the determinants of PNC services use in Enugu and reasons for defaults which this study aimed to assess [2, 12]. The findings of this study will help to improve PNC service utilization in Enugu. Knowledge on the determinants of postnatal care may also assist the policy makers to design, justify and implement appropriate interventions [5, 7].

This study aimed at determining the level of 6^th^ week postnatal care services utilization in Enugu and reasons for non-utilization as well as comparison between users and nonusers.

## Methods

### Study area and population

This study was conducted in the Institute of Child Health of both the University of Nigeria Teaching Hospital (UNTH) and Enugu State University Teaching Hospital (ESUTH) from June 2019 to September 2019. Both Institutes hold their immunization programs three times in a week. Women at various times after delivery bring their babies for immunization, even as early as first/second day of live and immunization of children can be extended up to 2 years of age. The women come from different parts of Enugu, irrespective of place of delivery and immunization is mostly free.

### Study design

It was a cross-sectional study of 400 consecutive women in their postpartum period who brought their babies for the second Oral Polio vaccine (OPV2) at 10 weeks postpartum.

### Inclusion and exclusion criteria

Mothers who came with their babies at 10 weeks for OPV2 immunization and gave their consent were included in the study However, women who did not give their consent and those who were not physically present at the immunization were excluded from the study. Postnatal care service utilization was measured as the use of postnatal care services by mothers following childbirth within 6 weeks irrespective of place of delivery.

### Sampling technique

As women were screened, the 6^th^ week PNC default case was recruited into the study group. The next woman, who attended 6^th^ week PNC, matched for parity group, was recruited into the control group. Convenience sampling method was used.

### Study variables

Variables such as place of antenatal and delivery, number of antenatal visits, type of antenatal care giver, complications during antenatal and delivery, mode of delivery, awareness of PNC, social class, marital status and age were compared between the 2 groups.

### Data collection

Interviewer-administered questionnaire was used for data collection. The data collected included socio-demographic characteristics of the participants, the history of postnatal care attendance and reasons for non-attendance, who gave healthcare during antenatal period as well as history of delivery and postpartum period. The questionnaire was pretested on 15 nursing mothers who were excluded from the study.

### Sample size calculation

The sample size (n) was determined using n = 2 x (Z_α_ + Z_β_)^2^ x P x (1-P) / (P_0_ –P_1_)^2^ [17], with Zα = 1.96 at 95% confidence level, power of 80% and 41.2% [16] as the utilization of postnatal services in Nigeria as well as 10% attrition rate, the calculated sample size was 150 in each arm. However, 400 women participated in the study.

### Ethical consideration

The ethical approval for the study was obtained from UNTH Research Ethics Committee (Ref. UNTH/CSA/329/Vol.5) and the research was carried out in accordance with the guidelines and regulations of the Ethics Committee. Informed and written consent was obtained and signed in the presence of a witness. However, parents or legal guardians consent was obtained for all participants under age of 18 years.

### Statistical analysis

Data was analyzed using the Statistical Package for the Social Sciences (SPSS) software, version 22.0, IBM SPSS, Chicago, Illinois. The results were presented in tables. Analysis was both descriptive and inferential at 95% confidence level. Chi square was used to analyze discreet variables and logistic regression where applicable. A p-value of less than 0.05 was considered statistically significant.

### Definition of terms

Postnatal attendance was defined as those women who attended postnatal clinic at the 6^th^ week postpartum.

Skilled birth attendant was defined as accredited health professional such as midwife, doctor or nurse who has been trained in the skills needed to manage pregnancies, childbirth and immediate postnatal period.

Primiparous was defined as those women with one child, Multiparous were those with 2–4 children while grand-multiparous were those with 5 or more children.

Social classification of the participants was as defined by Olusanya et al [18] which is based on education of the woman and her husband’s occupation as shown below:

### Social classification by Olusanya et al [18]

**Table:** 

A. Husband		B. Wife	
Score	Occupation	Score	Education
1	Professionals	0	University
2	Middle level	1	Secondary
3	Unskilled	2	Primary

Thus, the social class of each participant was then be determined by the sum of scores A and B to give Social classes I–V for example husband being a professional (1) and wife being a University graduate (0) gives Social Class I, etc

## Results

### Background characteristics of the participants

Four hundred women participated in the study.

The majority of the participants were aged 26–30 years (37.3%) and 31–35 years (35.8%) respectively. One hundred and sixty four (41%) of the women were salary earners. The majority of the participants were married (99.5%), Christians (100%), Igbo (95%), had tertiary level of education (72.3%) and multiparous (57.8%). Further details of the socio-demographic characteristics of the participants are as seen in **Table 1**.

**Table 1:** Socio-demographic characteristics of the participants.

	Frequency	Percent
***Age group***		
< = 25	58	14.5
26–30	149	37.3
31–35	143	35.8
36–40	42	10.5
41–45	8	2.0
***Occupation***		
Business/trader	151	37.8
Unemployed	48	12.0
Student	37	9.3
Salary earner	164	41.0
***Marital status***		
Married	398	99.5
Single	2	0.5
***Religion***		
Christianity	400	100.0
***Tribe***		
Igbo	380	95.0
Others	20	5.0
***Level of education***		
Primary	27	6.8
Secondary	84	21.0
Tertiary	289	72.3
***Parity***		
Primipara	152	38.0
Multipara	231	57.8
Grand multipara	17	4.3
***Socio-economic status***		
1	156	39.0
2	120	30.0
3	41	10.3
4	59	14.8
5	24	6.0

### Postnatal care awareness and attendance

Of the 400 women, 235 participants attended the 6^th^ week postnatal clinic (control group) while 165 women did not attend (study group). The 2 groups were comparable in terms of their socio-demographic characteristics except for their socio-economic status.

Although 74% (n = 296) of the women were aware of postnatal care, only 59% attended the 6 weeks postnatal clinic. The prevalence of non-attendance of the 6^th^ week postnatal clinic attendance among the mothers was 41% (N = 165).

### Comparison of postnatal clinic attendance of those who were attended to by skilled birth attendants and Non-skilled birth attendants

The majority of the women (60.6%) who had antenatal care by skilled birth attendants attended postnatal clinic while none of the women who were attended to by non-skilled birth attendants during antenatal attended postnatal clinic (*χ*^2^ = 17.619, p < 0.001). **Table 2.**

**Table 2:** Comparison of PNC attendance of those who were attended to by skilled birth attendants and Non-skilled birth attendants.

	Postnatal Clinic attendance			
Antenatal attendants	Yes	No	*χ* ^2^	P value
	n (%)	n (%)		
Skilled birth attendant	235 (60.6)	153 (39.4)	17.619	< 0.001
Non-skilled birth attendant	0 (0.0)	12 (100.0)		

### Comparison of the proportion of PNC defaulters that had at least 4 ANC visits to a skilled birth attendant during the last delivery and women that had PNC

Higher proportion of women who had at least 4 antenatal visits to skilled birth attendants made use of PNC services (**Table 3)**.

**Table 3:** Shows the proportion of PNC defaulters that had at least 4 antenatal clinic visits to skilled birth attendants during last delivery compared with that of women that had PNC.

Postnatal clinic attendance	Frequency	Percent
Yes	232	60.9
No	149	39.1

### Reasons for non-use of postnatal care services

The majority of the PNC defaulters were not aware of postnatal care (61.8%). Other reasons are as seen in **Table 4.**

**Table 4:** Reasons for non-use of postnatal care services among defaulters in Enugu, Nigeria.

	Frequency	Percent
Not aware of it	102	61.8
Being healthy	35	21.2
No time	13	7.9
Distant location of the hospital	4	2.4
Forgot	9	5.5
Poor attitude of birth attendants	2	1.2

### Comparison of socio-economic characteristics and postnatal clinic attendance

Socioeconomic status was significantly associated with postnatal clinic attendance (*χ*^2^ = 56.874, p < 0.001). Postnatal clinic attendance increased with increased socio-economic status. However, age and marital status were not significantly associated with postnatal care attendance (p > 0.05). **Table 5.**

**Table 5:** Association between socio demographic characteristics and postnatal clinic attendance.

	Postnatal clinic attendance			
	Yes	No	*χ* ^2^	P value
	n (%)	n (%)		
***Age group***				
≤25	29 (50.0)	29 (50.0)	5.985	0.200
26–30	82 (55.0)	67 (45.0)		
31–35	94 (65.7)	49 (34.3)		
36–40	26 (61.9)	16 (38.1)		
41–45	4 (50.0)	4 (50.0)		
***Marital Status***				
Married	233 (58.5)	165 (41.5)	1.411	0.235
Single	2 (100.0)	0 (0.00		
***Socioeconomic status***				
1	110 (70.5)	46 (29.5)	56.874	< 0.001
2	86 (71.7)	34 (28.3)		
3	15 (36.6)	26 (63.4)		
4	19 (32.2)	40 (67.8)		
5	5 (20.8)	19 (79.2)		
***Parity***				
Primipara	92 (60.5)	60 (39.5)	2.366	0.306
Multipara	136 (58.9)	95 (41.1)		
Grand multipara	7 (41.2)	10 (58.8)		

### Factors affecting postnatal clinic attendance

Women who attended antenatal in a hospital were 4 times more likely to attend postnatal clinic (OR = 3.706, 95% C.I = 2.105–6.524, p < 0.001). Women who had normal delivery were less likely to attend postnatal care (OR = 0.382, 95% C.I = 0.242–0.605, p < 0.001) when compared with those that had caesarean section. Postnatal care awareness was significantly associated with postnatal clinic attendance (*χ*^2^ = 200.164, P < 0.001). Hospital as a place of delivery was significantly associated with postnatal care attendance (*X*^2^ = 46.620, *p* < 0.001). Complications during pregnancy and delivery were significantly associated with postnatal clinic attendance (*χ*2 = 11.49, *p* = 0.009 and *X*^2^ = 9.707, *p* = 0.002 respectively). Exclusive breastfeeding was significant associated with PNC services use (*X*^2^ = 4.313, *p* = 0.038). Number of antenatal visits and the women’s perception about the delivery fees were not significantly associated with PNC services utilization. **Tables 6 and 7.**

**Table 6:** Shows association between other factors and postnatal clinic attendance.

	Postnatal clinic attendance			
	Yes	No	*χ* ^2^	P value
	n (%)	n (%)		
***Place of delivery***				
Hospital	217 (66.8)	108 (33.2)	46.620	P < 0.001
Health center	15 (23.8)	48 (76.2)		
Home	3 (30.0)	7 (70.0)		
Traditional birth attendant	0 (0.0)	2 (100.0)		
***Postnatal care awareness***				
Yes	235 (79.4)	61 (20.6)	200.164	P < 0.001
No	0 (0.0)	104 (100.0)		
***Complications after delivery***				
Bleeding	13 (65.0)	7 (35.0)	9.707	P = 0.002
None	0 (0.0)	8 (100.0)		
***Problems during antenatal***				
Hypertension	8 (66.7)	4 (33.3)	11.494	P = 0.009
Diabetes	2 (40.00	3 (60.0)		
Bleeding	13 (100.0)	0 (0.0)		
Others	2 (33.3)	4 (66.7)		
***Perception about delivery fees***				
Too much	72 (66.1)	37 (33.9)	3.586	0.166
Too little	4 (66.7)	2 (33.3)		
Just OK/Adequate	159 (55.8)	126 (44.2)		

**Table 7:** Shows association between other factors and postnatal clinic attendance (Contd.).

	Postnatal clinic attendance				
	Yes	No	OR	95% C.I for OR	P value
	n (%)	n (%)			
***Place of antenatal***					
Hospital	214 (63.9)	121 (36.1)	3.706	2.105–6.524	< 0.001
Health center	21 (32.3)	44 (67.7)			
***Number of antenatal visits***					
1–3 times	3 (42.9)	4 (57.1)	0.520	0.115–2.357	0.397
4 or more times	232 (59.0)	161 (41.0)			
***Mode of delivery***					
Normal delivery	140 (51.7)	131 (48.3)	0.382	0.242–0.605	< 0.001
Caesarean section	95 (73.6)	34 (26.4)			
***Exclusive breast feeding***					
Yes	175 (62.1)	107 (37.9)	1.581	1.025–2.439	0.038
No	60 (50.8)	58 (49.2)			

After including the factors in a multivariate model, place of antenatal (OR = 2.870, 95% C.I = 1.590–5.180, p < 0.001) and mode of delivery (OR = 0.452, 95% C.I = 0.280–0.728, p = 0.001) were the only significant predictors of postnatal clinic attendance (p < 0.05). Table 8.

**Table 8:** Multivariate analysis of factors associated with postnatal clinic attendance.

	OR	95% C.I for OR	P value
Place of antenatal (hospital)	2.870	1.590–5.180	< 0.001
Number of antenatal visits (1–3 times)	0.990	0.198–4.944	0.990
Mode of delivery (normal delivery)	0.452	0.280–0.728	0.001
Exclusive breast feeding (yes)	1.569	0.994–2.477	0.053

## Discussion

Despite the importance of postnatal care, its awareness and utilization have been very poor. Although the majority of the participants in our study were aware of postnatal care, only 59% of the women utilized PNC services. Though the prevalence of PNC utilization obtained in our study was higher than in other similar studies done in Nigeria [7, 13, 16]; higher prevalence of 74.4% - 89.2% also had been reported [5, 19]. This difference may be attributed to the study subjects and the setting. Our study was conducted in 2 tertiary hospitals in Enugu and the majority of the women had tertiary level of education and of high socioeconomic status. Women with higher level of educational attainment are more likely to seek information about safe motherhood and newborn care. The study subjects and setting may also explain why almost the same prevalence (52.5%) was obtained from a similar study done in Enugu [8].

Women who were attended to by skilled birth attendants during their antenatal care were more likely to attend postnatal clinic. This may be because the skilled birth attendants would have counselled the women about the importance of PNC services use. Also during health talks given by the nurses/midwives during antenatal visits; women are taught many things including the importance of postnatal clinic attendance. This study finding was in agreement with previous related studies [20, 21].

Women who were aware of postnatal care were more likely to utilize the services than those who never heard about it. This is because those who were aware of PNC were more likely to know the benefits of its utilization and the health risks during postpartum period. This finding was consistent with previous studies [5, 13, 19, 22]. Furthermore, majority of women who defaulted from PNC attendance were not aware of it and most of them were attended to by non-skilled birth attendants during their antenatal period. However, some women who were aware of PNC did not utilize it because they felt they were healthy and therefore not necessary to attend PNC. Knowledge of PNC alone may not be the only factor necessary for PNC utilization but increase awareness can definitely be beneficial.

Our study showed that socioeconomic status was significantly associated with PNC utilization. This may be attributed to the fact such women had higher level of exposure to knowledge and better health care during their pregnancies. This finding was in agreement with similar study [5, 9].

Women who delivered in a hospital were more likely to utilize PNC services compared to those who delivered outside the hospital. Such women were more likely to get health education and counselling regarding PNC services. This finding was similar to what was obtained in related studies [5, 22].

Mode of delivery was significantly associated with postnatal clinic attendance. Women who had normal delivery were less likely to make use of the PNC services compared with women who had caesarean section. This is because women who had caesarean section would want to ensure that the wound healed perfectly while one who had normal delivery would assume she is healthy and therefore decide that going for postnatal care was unnecessary. This finding was similar to related studies [1, 11].

Women who had complications such as bleeding during pregnancy or delivery were more likely to utilize PNC services. Such women need reassurance that their health has been restored.

Exclusive breastfeeding of the newborn increased the chance of PNC services utilization. This may be because mothers practicing exclusive breastfeeding may want discuss breast care, nutrition and more information on breastfeeding.

### Strengths and limitations

A relatively large sample size was used and trained interviewer administered the questionnaires. Also the study was conducted in two immunization centers in Enugu.

This study had some limitations. Although the study was conducted at 10 weeks postpartum period, there may have been some recall bias. Also data collected were based on self-report and may not have reflected the opinions of the respondents. These limitations were minimized by the use of interviewers who were trained for the study.

### Recommendations

We recommend the following: (1) Health education and counselling regarding PNC services should be given more priority. Awareness should be created even on social media and other means of communication. (2) Women in Enugu should be encouraged to have their antenatal care under skilled birth attendants. This will increase PNC services utilization and therefore improve maternal and child health. (3) Government should make health care available and more affordable for women in order to prevent them from patronizing unskilled birth attendants.

## Conclusion

Postnatal clinic attendance by women in Enugu is still suboptimal. The main reason for non-attendance of the 6^th^ week postnatal clinic is because of lack of awareness. There is need for healthcare professionals to create awareness about the importance of postnatal care and encourage mothers to attend. A larger study involving more immunization centers in Enugu and Nigeria will be needed to generalize the result to the general population especially the ones from different study population.

## Supporting information

S1 File (TXT)
